# XUV-driven plasma switch for THz: new spatio-temporal overlap tool for XUV–THz pump–probe experiments at FELs^1^

**DOI:** 10.1107/S1600577519014164

**Published:** 2020-01-01

**Authors:** E. Zapolnova, R. Pan, T. Golz, M. Sindik, M. Nikolic, M. Temme, M. Rabasovic, D. Grujic, Z. Chen, S. Toleikis, N. Stojanovic

**Affiliations:** a Deutsches Elektronen-Synchrotron (DESY), Notkestrasse 85, 22607 Hamburg, Germany; b Institute of Physics Belgrade, Pregrevica 118, 11080 Belgrade, Serbia; c SLAC National Accelerator Laboratory, Menlo Park, CA 94025, USA

**Keywords:** plasma switch, XUV, pump–probe, temporal overlap

## Abstract

A simple and robust tool for spatio-temporal overlap of THz and XUV pulses in in-vacuum pump–probe experiments is presented. The technique exploits ultrafast changes of the optical properties in semiconductors (*i.e.* silicon) driven by ultrashort XUV pulses that are probed by THz pulses. This work demonstrates that this tool can be used for a large range of XUV fluences that are significantly lower than when probing by visible and near-infrared pulses. This tool is mainly targeted at emerging X-ray free-electron laser facilities, but can be utilized also at table-top high-harmonics sources.

## Abstract 2

A THz plasma switch, driven by ultrafast XUV pulses for temporal ovelap in pump–probe experiments, is presented.

## 1. Introduction   

Intense THz pulses combined with synchronized X-ray pulses enable investigation of the dynamics of the light–matter interaction, non-linear response of materials and control of the properties of matter selectively on femtosecond time scales. Therefore, achieving the temporal overlap between pump and probe pulses in the femtosecond range is essential. Certain pump–probe schemes, *e.g.* THz streaking (Frühling *et al.*, 2009 ▸; Schmid *et al.*, 2019 ▸), are comparatively tolerant against the spatial overlap between XUV and THz pulses and the actual focal position of the THz beam. The observable, *i.e.* the kinetic energy of the photoelectrons, is furthermore of considerable magnitude and can be utilized for further optimization of the pump–probe signal. This is almost never the case in pump–probe experiments on solid-state samples, utilizing one of the XUV probing techniques [*e.g.* X-ray magnetic circular dichroism (XMCD) (Pfau *et al.*, 2012 ▸; Willems *et al.*, 2015 ▸) and resonant inelastic X-ray scattering (Dell’Angela *et al.*, 2016 ▸)]. There, the spatio-temporal overlap between THz and XUV and in particular diffraction-limited focusing of the THz beam have to be achieved with the aid of versatile in-vacuum diagnostics.

The so-called plasma-switch, the transient change of optical constants in the visible (VIS) and near-infrared (NIR) spectral ranges by X-ray and XUV pulses, has been used for the temporal characterization of these pulses (Harmand *et al.*, 2012 ▸; Gahl *et al.*, 2008 ▸; Krupin *et al.*, 2012 ▸; Riedel *et al.*, 2013 ▸; Danailov *et al.*, 2014 ▸). Transient changes of optical properties in the THz range, driven by femtosecond laser pulses, have been used for pickup of individual pulses from MHz trains at infrared free-electron lasers (FELs) (Schmidt *et al.*, 2015 ▸) as well as for THz spectral shaping at table-top THz sources (Cartella *et al.*, 2014 ▸; Mayer *et al.*, 2014 ▸).

**Figure 1:**
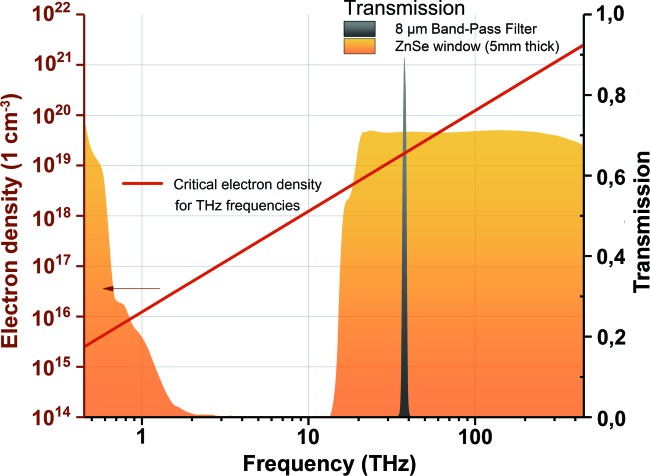
Calculation of the critical electron density for the THz range (red line). Transmission of the 5 mm-thick ZnSe vacuum window and the bandpass filter (at 8 µm wavelength) used in the experiment are presented as the shadowed areas.

As shown in Fig. 1 ▸, for lower probing frequencies the effect of the plasma switch is more efficient because lower electron density is required to change the material reflectivity. In this work we present a technique to establish the temporal overlap between XUV and THz pulses, based on the transient change of optical properties of a silicon target in the THz spectral range, induced by the intense femtosecond XUV pulse.

The presented method can be applied in facilities employing THz radiation for time-resolved XUV–THz pump–probe experiments where it is necessary to temporally overlap XUV and THz pulses on a sub-picosecond level.

## 2. XUV driven THz plasma switch: theoretical background   

The process of electronic excitation of materials by an intense XUV pulse happens on an ultrafast time scale, within a few femtoseconds (Gahl *et al.*, 2008 ▸; Riedel *et al.*, 2013 ▸), and is governed by the photoionization of the electrons in the material: photoabsorption of the bound electrons within the valence band, secondary processes as elastic and inelastic scattering of free electrons, Auger decay, and electron pair creation. Other processes may contribute to the photoionization depending on the energy of the incoming photon and the material (Medvedev & Rethfeld, 2010 ▸). Previous theoretical studies have shown that the density of the created free electrons follows the photon flux of the XUV pulse linearly (Riedel *et al.*, 2013 ▸) in a wide intensity range, below fluences required for the sample melting, ablation and plasma formation.

Optical properties of the photo-excited material strongly depend on the density of free electrons and can be modelled [*e.g.* via the continuity equation (Mezentsev *et al.*, 2007 ▸)] and expressed in terms of relative permittivity. According to the Drude model, free electrons in a material can be treated as free-electron plasma with a corresponding plasma frequency ω_p_ (Ashcroft & Mermin, 1976). We assume that the damping can be neglected in our case (refer to Appendix *A* [App appa] for a short discussion on this topic) and the relative permittivity ∊ in this case can be presented as a function of the incoming frequency ω and the plasma frequency ω_p_,
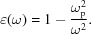
This indicates that light with a higher frequency than the plasma frequency, ω > ω_p_, can penetrate the plasma whereas light with lower frequency, ω < ω_p_, will be reflected. Taking into account the oscillatory motion of the electron, the critical electron density, *n*_e_, required to make the sample reflective to light with a certain frequency can be presented as
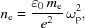
where ∊_0_ is the vacuum permittivity, *e* is the charge and *m*_e_ is the mass of an electron.

In our experiment, the critical electron density for the probing pulse at a wavelength of 8 µm (37.5 THz) is *n*_e 8µm_ = 1.8 × 10^19^ cm^−3^, and at wavelengths over 100 µm (<3 THz) it is less than *n*_e 100µm_ = 1.1 × 10^17^ cm^−3^.

## 3. Description of the setup   

The experiment was performed with the pump XUV wavelength at 13.5 nm (91.8 eV) and two different probing conditions: (i) a THz pulse with a central wavelength of 8 µm, and (ii) a broadband THz pulse with a wavelength >100 µm. The expected pulse duration for THz was ∼300 fs and ∼3 ps, respectively, and the XUV pulse duration was 160 fs, estimated by electron bunch length measurements by a transverse deflecting RF-structure (Düsterer *et al.*, 2014 ▸).

**Figure 2:**
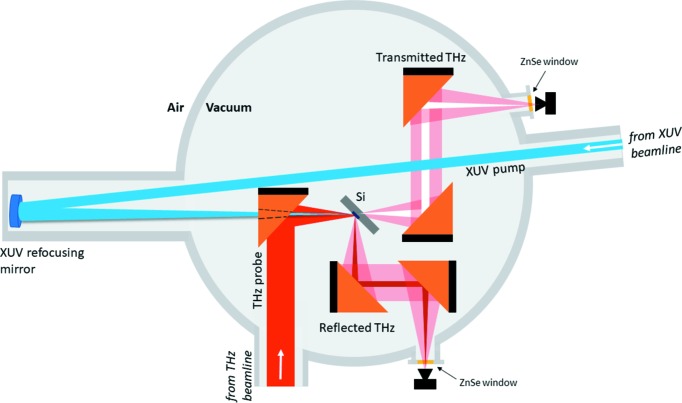
Scheme of the XUV-driven plasma switch experiment for the THz beam. The THz and XUV beams are collinearly focused and spatially overlapped on a 400 µm-thick Si sample at a 45° incidence angle. Transmitted and reflected THz beams are picked up by off-axis parabolic mirrors and further focused on the corresponding pyro detectors through 5 mm-thick ZnSe vacuum windows.

The THz beam is collimated using five toroidal mirrors in order to keep the beam size within the range of the beam transport and optics. This additional folding of the THz beam results in a ∼6.5 m longer optical path with respect to the XUV beam. In order to overlap the XUV and THz pulses in time, an additional delay for the XUV is introduced: pulses travel 3.25 m longer distance and then are refocused by a mirror with 3.5 m focal length back to the experiment (Pan *et al.*, 2019 ▸). The scheme of the experiment is presented in Fig. 2 ▸.

The THz and XUV pulses are collinearly focused and spatially overlapped in the experimental chamber on a 400 µm-thick Si sample at a 45° incident angle. The transmitted and reflected portions of the THz beam are picked up and collimated using parabolic mirrors. Then they are focused through ZnSe vacuum windows (5 mm thick) on two 2 mm × 2 mm pyro detectors (InfraTec LME-301) located outside of the experimental chamber in air ∼5 mm from the window. The detectors were custom-designed by collaboration of the DESY FLA group and InfraTec to reduce internal THz interferences (Wesch, 2012 ▸). The detectors are without optical windows, which makes them suitable for measurements along a broad spectral range and sensitive to XUV radiation. ZnSe vacuum windows have good transmission in the VIS to IR range as well as in the long-THz wavelength range (see Fig. 1 ▸).

Pulse energies of the XUV, measured with the gas-monitor detector (GMD), were 110 µJ ± 20 µJ (r.m.s.) (Tiedtke *et al.*, 2009 ▸) and 0.5 µJ ± 0.1 µJ (r.m.s.) for the THz beam measured with a calibrated pulse energy meter (Zapolnova *et al.*, 2018 ▸; Pan *et al.*, 2019 ▸).

The estimated XUV pulse energy through the beamline (Tiedtke *et al.*, 2009 ▸) after the refocusing mirror and through attenuation filters was 700 nJ ± 10 nJ (refer to Table 1 ▸ for details), yielding a final intensity on the sample of 6.76 × 10^9^ W cm^−2^ and 2.65 × 10^9^ W cm^−2^, for the two measured XUV beam sizes (see Section 4 for details). By measuring both transmitted and reflected intensities of the THz beam and assuming that absorption in the excited Si layer is negligible, we are able to correct the pulse-to-pulse energy fluctuations of the THz beam (3.6% RMS at 100 µm, 14% RMS at 8 µm).

## 4. THz and XUV 2D beam profile   

The THz and XUV beams were characterized by 2D profile measurements in the focal position. A pyro detector with a 100 µm pinhole was mounted on an *xy* positioner, facing the incoming THz and XUV beams at normal incidence, and was moved through the focus of the beam with defined steps along the *z* axis. The pyro detector also showed a good response for XUV radiation, and therefore it was used for both the THz and XUV beam profile characterizations.

**Figure 3:**
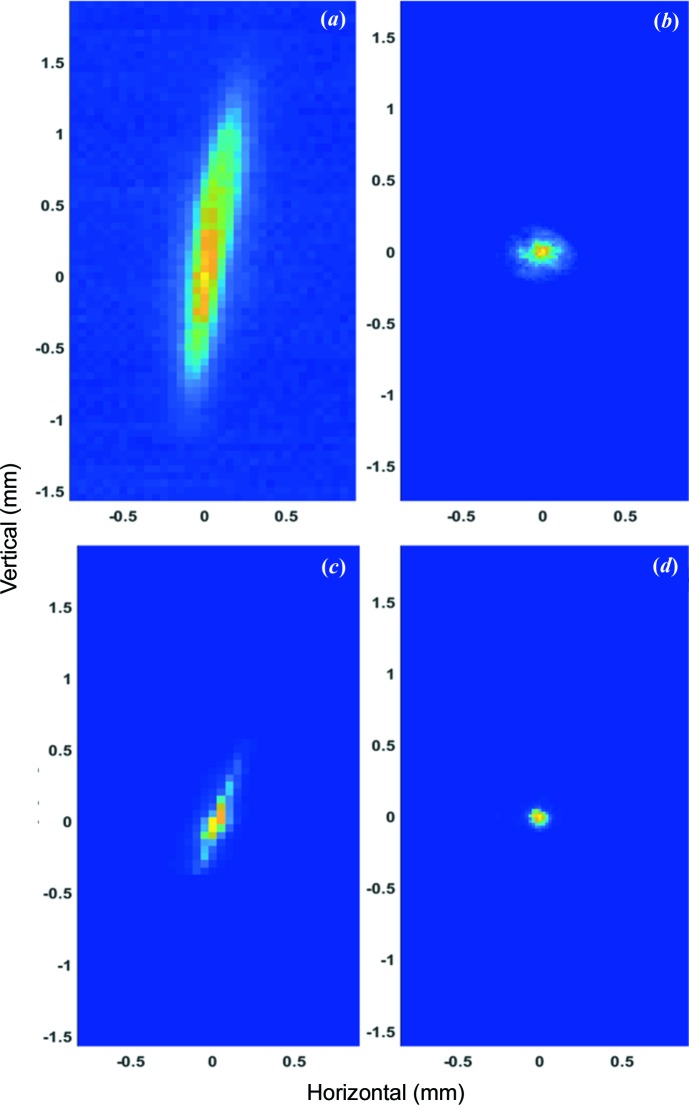
Measured 2D profiles of the THz and XUV beams. (*a*) THz beam profile at 100 µm with an FWHM of 400 ± 20 µm × 1470 ± 30 µm. (*b*) XUV beam at 13.5 nm wavelength through a 3 mm pinhole placed ∼30 m upstream of the experiment with an FWHM of 230 ± 30 µm. (*c*) THz beam profile at 8 µm wavelength with an FWHM of 180 ± 15 µm × 320 ± 15 µm. (*d*) XUV beam at 13.5 nm wavelength with a 10 mm pinhole at the same position as in (*b*) with an FWHM of 140 ± 20 µm.

The results of 2D scans are presented in Fig. 3 ▸. The THz beam in focus has an ellipsoidal profile, elongated in the vertical direction, because of imperfect alignment of the off-axis parabolic mirror for the THz beam. The full width at half-maximum (FWHM) diameter of the THz beam with the THz undulator set at a 100 µm nominal wavelength was 400 ± 20 µm × 1470 ± 30 µm, and at 8 µm it was 180 ± 15 µm × 320 ± 15 µm. In an attempt to match the XUV and THz beam sizes we inserted a pinhole (3 mm diameter) in the XUV beam, 30 m upstream of the experiment, to optimize the ratio between beam sizes. The FWHM diameters of the XUV beam with and without a pinhole were 230 ± 30 µm and 140 ± 20 µm, respectively. The ratio between the areas of the THz and XUV beams was 1:9 for the THz beam at 100 µm and 2:3 at 8 µm.

## 5. Transient reflectivity and transmission   

**Figure 4:**
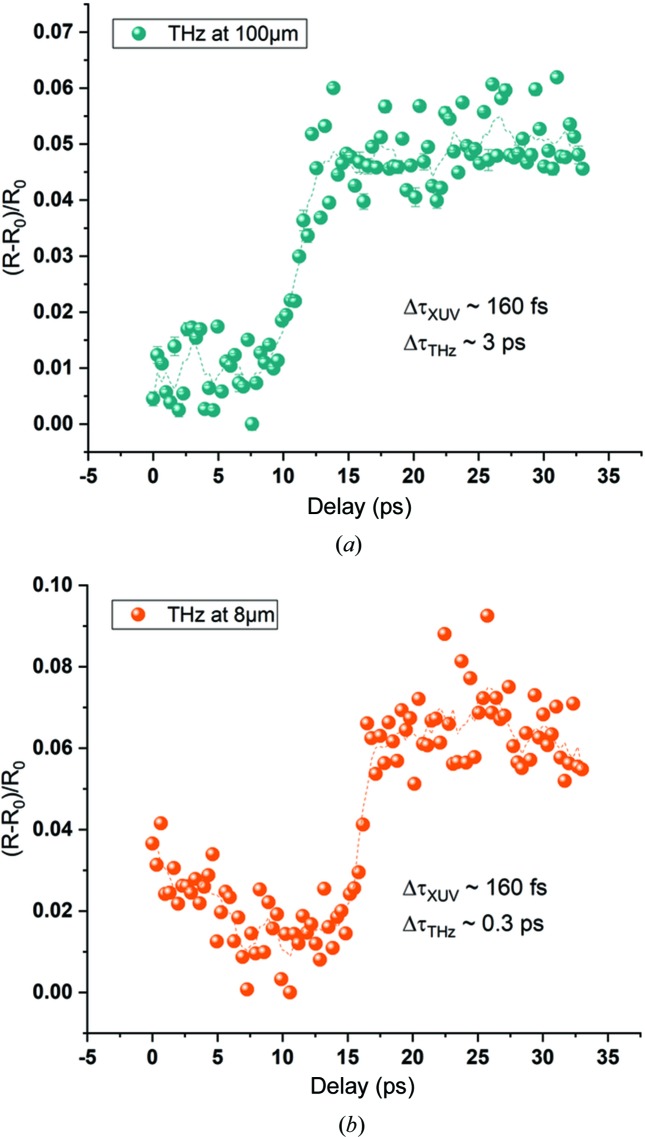
Transient optical reflectivity curves for the THz undulator set at 100 µm and 8 µm wavelengths for a 13.5 nm XUV pump wavelength.

Results of time-dependent reflectivity measurements [presented as (*R* − *R*_0_)/*R*_0_, where *R*_0_ is the equilibrium reflectivity] are presented in Fig. 4 ▸. Once the probing THz pulse arrives following the XUV pulse, a portion of the THz pulse, which spatially overlaps with the XUV pulse, is reflected more because of the plasma created by the XUV pulse. The observed duration of the transition (slope) Δτ_λ_THz__ is the convolution of the pulse durations of the THz Δτ_THz_ and XUV pulses Δτ_XUV_, the jitter Δτ_jitter_ between them, and the timescale of the free carrier excitation process Δτ_excitation_, and can be described as 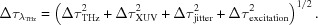
For a THz wavelength of ∼100 µm, the observed slope width is Δτ_100µm_ = 2.2 ps and for 8 µm wavelength it is Δτ_8µm_ = 1.2 ps (calculated as the time between the points corresponding to the 10% and 90% levels of total amplitude of the signal). The XUV and THz pulses are naturally synchronized in this experiment, with jitter smaller than 5 fs (RMS) (Frühling *et al.*, 2009 ▸), and its contribution is negligible. We assume that the excitation of the free carriers is much faster than other timescales in the experiment so we neglect it as well.

## 6. Dependence on the XUV fluence   

**Figure 5:**
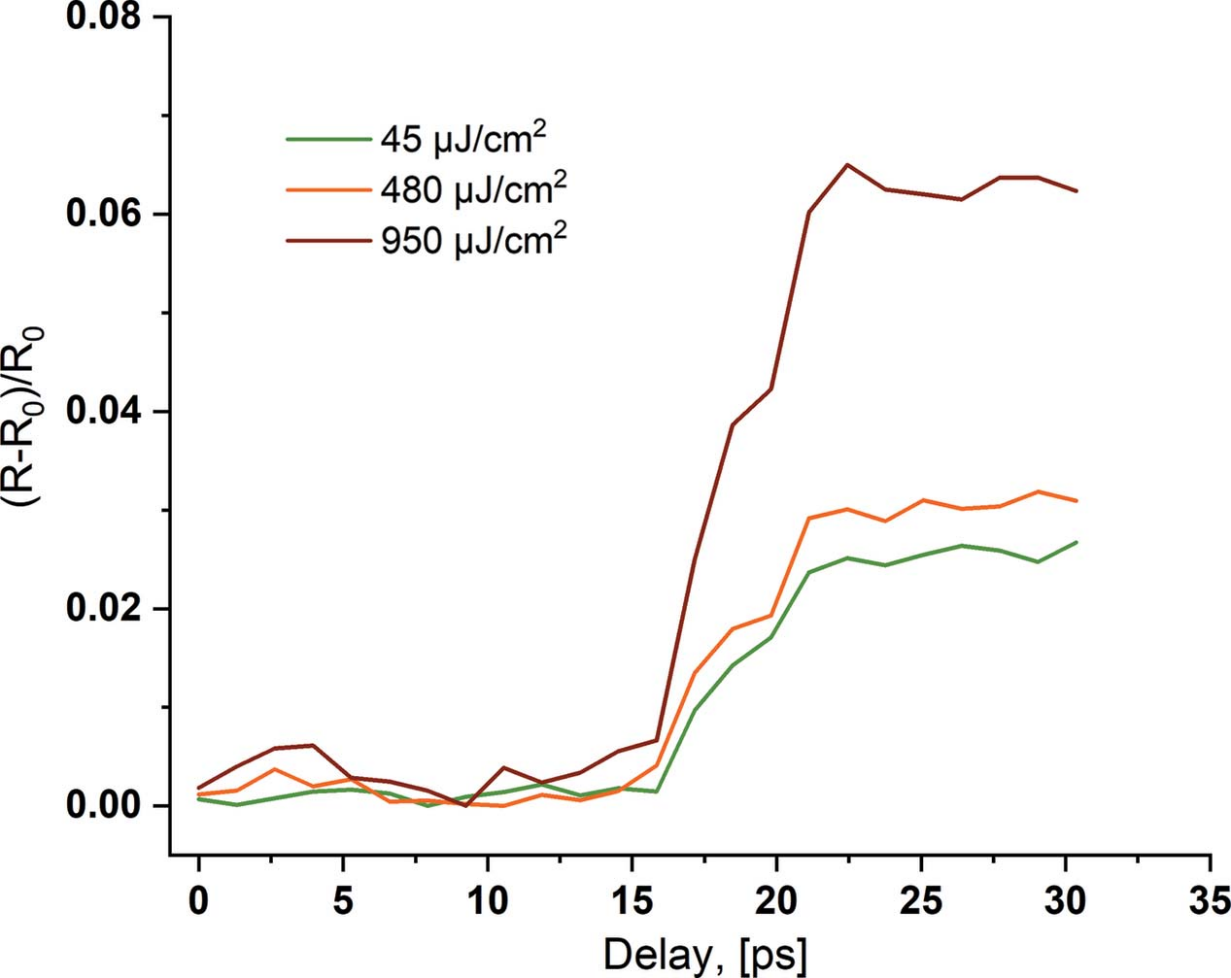
Transient THz reflectivity curves as a function of the THz/XUV pulse delay for three different fluences of the XUV pulse on the sample.

Fig. 5 ▸ shows a comparison of the transient THz reflectivity change for different fluences of the pump XUV pulse. We used different combinations of the attenuation filters: Si_3_N_4_ 350 nm (red line), Si_2_N_4_ 350 nm + Nb 405 nm (orange line) and Si_3_N_4_ 500 nm (green line). The effect of the plasma switch in the THz spectral range is very efficient and can be clearly observed even at XUV fluences as low as 45 µJ cm^−2^.

## 7. Quantitative estimate of the effect   

The amplitude of the reflectivity change for a broadband THz beam >100 µm is around 6.4% and for 8 µm is around 6.0%. Using the details of the actual THz and XUV beam sizes from the 2D profile measurements, we can estimate the actual switched fraction of the THz pulse. Comparing total areas of the beams and assuming that the electron density follows the intensity envelope of the XUV beam linearly, we can assume that, if the XUV beam size matches the size of the THz beams for a 100 µm wavelength (400 µm FWHM beam size) and for a 8 µm wavelength (180 µm FWHM beam size), the overall effect on the reflectivity change would be 9 times higher (∼57.6%) and 1.5 times higher (∼10%) than observed.

## 8. Summary   

We have developed a tool for temporal and spatial overlap of XUV and THz pulses in pump–probe experiments, based on an XUV plasma switch for the THz range on an Si sample. During several pump–probe experiments at FLASH, it was demonstrated that the arrival time of XUV and THz pulses can be established down to at least the pulse duration of the THz pulse.

The experiment has been performed at different XUV fluences from 0.045 mJ cm^−2^ up to 0.95 mJ cm^−2^ for 8 µm wavelength and for the broadband >100 µm wavelength of the probe pulse. The observed change of the transient normalized reflectivity (*R* − *R*_0_)/*R*_0_ of THz beam due to the plasma switch is approximately 6% from the initial level.

Since this effect uses low XUV fluences, far below the damage threshold, and uses room-temperature broadband THz detectors, it is robust and simple. This technique can be further applied at facilities employing XUV–THz pump–probe experiments, and enables a straightforward and efficient method for temporal overlap of XUV and THz pulses on the picosecond time scale.

## Figures and Tables

**Table 1:** Transmission of XUV at 13.5 nm

XUV pulse energy via GMD	112 µJ ± 17 µJ
Beamline transmission	78%
Refocusing mirror	62%
Si_3_N_4_ 500 nm filter transmission	1.3%
Total transmission	700 nJ ± 10 nJ

## Appendix A Comparison of the excited-layer thickness with the penetration depth of THz radiation

The frequency-dependent dielectric constant, according to the simple Drude model, where damping is independent of the free electron energy, can be expressed as (Van Exter & Grischkowsky, 1990 ▸)

where ω_p_ is the plasma frequency, Γ = 1/τ_c_ is the damping frequency and τ_c_ is the average free-electron collision time. From the literature, we estimate the average free-electron collision time to be between 1 fs and 100 fs (Ashcroft & Mermin, 1976 ▸; Temnov *et al.*, 2006 ▸; Van Exter & Grischkowsky; 1990 ▸; Riedel *et al.*, 2013 ▸). Finally, this gives us the estimated minimum penetration depth for probing THz frequencies (2–40 THz) in XUV-excited plasma in silicon to be ≥2 µm.

For an XUV wavelength of 13.5 nm impinging at a 45° angle of incidence, the thickness of the excited area in silicon is 400 nm, as determined by the penetration depth (Henke *et al.*, 1993 ▸). The XUV pulses from FLASH, used in this work (as presented in Fig. 5 ▸), result in free-electron densities in the range from 1.2 × 10^17^ cm^−3^ up to 2.8 × 10^18^ cm^−3^.

This leads to the conclusion that only a small fraction of the probing THz radiation (<20%) is absorbed in the investigated sample excited by the XUV pulse.
